# Predictability of phases and magnitudes of natural decadal climate variability phenomena in CMIP5 experiments with the UKMO HadCM3, GFDL-CM2.1, NCAR-CCSM4, and MIROC5 global earth system models

**DOI:** 10.1007/s00382-018-4321-1

**Published:** 2018-06-27

**Authors:** Vikram M. Mehta, Katherin Mendoza, Hui Wang

**Affiliations:** 1 Center for Research on the Changing Earth System, 5523 Research Park Drive, Suite 205, Catonsville, MD 21228 USA; 2 Present Address: NOAA/Climate Prediction Center, College Park, MD USA

**Keywords:** Decadal climate variability, Climate predictability, Pacific decadal oscillation, Volcanic eruptions

## Abstract

Data from decadal hindcast experiments conducted under CMIP5 were used to assess the ability of CM2.1, HadCM3, MIROC5, and CCSM4 Earth System Models (ESMs) to hindcast sea-surface temperature (SST) indices of the Pacific Decadal Oscillation (PDO), the tropical Atlantic SST gradient (TAG) variability, and the West Pacific Warm Pool (WPWP) SST variability from 1961 to 2010. The ESMs were initialized at specific times with observed data to make 10- and 30-year hindcasts/forecasts. Deterministic and probabilistic skill estimates show predictability of detrended WPWP index to 5 years’ lead time and of non-detrended WPWP index to 10 years’ lead time. These estimates also show atypical skill dependence of PDO and TAG indices on lead times, with increasing skill in the middle to end of 10-year hindcasts. The skill of ESMs to hindcast an observed DCV index (signal skill) is surprisingly greater than the skill to hindcast their own DCV index (noise skill) at some lead times. All ESMs hindcast occurrence frequencies of positive and negative phases of the indices, and probabilities of same-phase transitions from one year to the next reasonably well. Four, major, low-latitude volcanic eruptions are associated with phase transitions of all observed and some of the ensemble-average hindcast indices. All ESMs’ WPWP index hindcasts respond correctly to all eruptions as do three observed PDO phase transitions. No one of the ESMs’ hindcasts of the TAG index responds correctly to these eruptions. Some of the ESMs hindcast correct phase transitions in the absence of eruptions also, implying that initializations with observed data are beneficial in predicting phase transitions. The skills of DCV indices’ phase prediction up to at least two years in advance can be used to inform societal impacts adaptation decisions in water resources management and agriculture. The Atlantic region’s responses in these ESMs appear to be fundamentally incorrect.

## Introduction

Among natural decadal climate variability (DCV) phenomena, the Pacific climate variability generally known as the Pacific Decadal Oscillation (PDO; Mantua et al. 1997) or the Inter-decadal Pacific Oscillation (IPO; Power et al. 1999), the tropical Atlantic sea surface temperature (SST) gradient (TAG; Hastenrath 1990; Houghton and Tourre 1992; Mehta and Delworth 1995; Mehta 1998; Rajagopalan et al. 1998), variability of the West Pacific Warm Pool (WPWP) SST (Wang and Mehta 2008), and their impacts on global climate are attracting increasing attention in predictability and prediction studies because of their impacts on water resources, agriculture, hydro-electricity generation, inland water-borne commerce, and fish and crustacean stocks and captures (Mehta 2017). Analyses of associations between SST indices of these three natural DCV phenomena; and decadal–multidecadal variability of global precipitation, temperatures, and the Palmer Drought Severity Index show that approximately 60–90% variance in these three hydro-meteorological variables on land is explained by the PDO, the TAG SST variability, and the WPWP SST variability (Mehta 2017).

Some fundamental and substantial problems of decadal climate prediction are (Meehl et al. 2009, 2014; Mehta et al. 2011a): (1) relatively short time series of instrument-based global ocean observations, especially sub-surface observations, for understanding, model initialization, and comparison with prediction; (2) an insufficient understanding of fundamental physics of DCV; (3) an insufficient theoretical understanding of possible behaviors of geographically-varying, complex and non-linear dynamical systems with mixed initial and boundary values; (4) global climate models displaying less than satisfactory skill in simulating climate in general and DCV in particular; and (5) insufficient guidance from stakeholders and policymakers as to which DCV-related climate, weather, and impacts information would be useful for applications to societal impacts of DCV if predicted. In spite of these problems, however, there have been many encouraging decadal prediction studies with global earth system models (ESMs). In these pioneering studies, ESMs were initialized from observed data, and natural and anthropogenic changes in aerosol optical depth (AOD) were prescribed from observations-based estimates (or scenarios). Smith et al. (2007) showed that skillful decadal prediction of global-average temperature may be possible. Keenlyside et al. (2008) and Pohlmann et al. (2009) showed that skillful prediction of decadal, North Atlantic SSTs may be possible. Building on these studies, Yang et al. (2012) found that an inter-hemispheric, multidecadal SST pattern in the Atlantic may be predictable 4 to 10 years in advance. Meehl et al. (2014) have described results from hitherto published CMIP5 and other decadal hindcasting experiments, so only major results pertaining to predictability of indices of decadal SST variability are reviewed here.

There have been two types of assessments of prediction skill of the PDO index; one, correlation coefficient between observed and predicted indices or area-average SSTs over several decades, and two, prediction skill of specific warm or cold events. An example of the former type is a skill assessment of decadal hindcasts of the PDO index in five CMIP5 ESMs by Kim et al. (2012) who found that there was a reasonably significant prediction skill for up to five years after prediction initialization, but that this skill was less than that derived from persistance of the PDO index. An example of the latter type of skill assessment is the improved prediction skill of the mid-to-late 1970s change in the PDO phase from negative (cold) to positive (warm) in combined initial and boundary value experiments with several CMIP5 and other ESMs by Meehl and Teng (2012, 2014) compared to uninitialized experiments or simulations as boundary value experiments. As mentioned earlier and described in detail by Meehl et al. (2014), reasonably high skill of area-average North Atlantic SSTs is shown by several ESMs [see, for example, Keenlyside et al. (2008), Pohlmann et al. (2009), van Oldenborgh et al. (2012), Yang et al. (2012), Hazeleger et al. (2013), Ham et al. (2014), and others]. Using decadal hindcast data from four CMIP5 ESMs, Mehta et al. (2013b) found that there was significant, but variable, decadal hindcast skill of global- and tropical ocean basin-average SSTs during 1961 to 2010. The skill varied by averaging region and decade. It was also found that low-latitude volcanic eruptions can be one of the sources of decadal SST hindcast skill when major eruptions occurred. In the four ESMs, decadal hindcast skills of SST anomalies over ocean basin size averaging regions generally improved due to model initialization with observed data. Kirtman et al. (2013) summarize conclusions about decadal prediction that “Predictions for averages of temperature, over large regions of the planet and for the global mean, exhibit positive skill when verified against observations for forecast periods up to ten years.” Thus, there is slow and incremental, but definite, progress in making skillful decadal climate predictions.

To expand the potential for applying decadal climate predictions to societal impacts adaptation, new prediction approaches need to be evolved from the points of view of users of decadal climate information—farmers, water managers, and other stakeholders and policymakers—if the predicted information is to be useful for application. Although impacts of quantitative changes in DCV indices on hydro-meteorology (and, consequently, on water resources and agriculture) have not attracted much attention from researchers, impacts of DCV phases—positive and negative—are known much better via analyses of empirical data and via experiments with numerical models of the global atmosphere [see, for example, Schubert et al. (2004a, b)]. Therefore, data and information such as phase (positive or negative) of average anomaly in precipitation and temperature, river flow, drought index, and other quantities over the next 2–10 years can be very useful for management decisions in water and agriculture sectors if the data and information are provided at the spatial resolution required for each sector (Mehta et al. 2013a; Mehta 2017). A study of the value of decadal climate information to the agriculture sector in the Missouri River Basin—the largest river basin in the U.S. and a major “bread basket” of the world - with a water and crop choices model showed that the correct prediction of important DCV phenomena one year in advance can be worth approximately $80 million per year (Fernandez et al. 2016). This study also showed that the correct prediction of even the phase of the DCV phenomena one year in advance can realize a sizeable fraction of this monetary value. Therefore, accurate prediction of DCV phase transitions sustained for several months to a year or longer can be useful in the adaptation of worldwide agriculture and water resources to DCV-related hydro-meteorological conditions, with important consequences for water and food securities. Since decision processes in these sectors utilize probabilistic information, accurate predictions of DCV phase transition probabilities would be very useful to these sectors. Understanding and prediction of DCV phases is also important for attribution of DCV phase transitions to internal ocean–atmosphere processes or changes in external forcings.

Another reason to evolve different approaches for decadal climate prediction is that, unlike in weather prediction, variations/changes in external or boundary forcings such as solar emissions, volcanic and anthropogenic aerosols, anthropogenic greenhouse gases, and land use—land cover also influence/impact climate at the multiyear to decadal timescales. Since decadal predictions using dynamical models are made as a mixed initial—boundary value problem, contributions of both model initialization and external/boundary forcings in decadal prediction skill should be evaluated. Therefore, comparison of initialized predictions with uninitialized simulations with the same ESMs is very important.

Based on the foregoing rationale, the ability of the CM2.1, HadCM3, MIROC5, and CCSM4 ESMs in CMIP5 to simulate major attributes of the PDO, the TAG variability, and the WPWP variability was described in Mehta et al. (2017). The ability of these four ESMs to hindcast the three DCV phenomena is addressed in the present paper. The scientific objectives of this study are: (1) to assess deterministic and probabilistic skills of these ESMs to hindcast the phases and magnitudes of the three DCV indices; (2) to assess transition probabilities of phases of the PDO, TAG, and WPWP indices, individually as well as in combinations of indices, and compare them with transition probabilities of observed indices; (3) to understand the roles of volcanic eruptions and internal ocean–atmosphere variability in predictability of phase transitions of DCV indices; and (4) to assess the impacts, if any, of initialization on hindcast skill.

These four ESMs were selected for both studies because it is important to assess simulation and hindcast skills of the same ESMs in the same experimental framework. The modeling groups who have developed these four ESMs conducted CMIP5 simulation and hindcast/forecast experiments with generally the same model configurations. Also, decadal hindcast/forecast experiments with these four ESMs were run in CMIP5 in the ensemble mode with up to 10 members in each ensemble.

## Materials and methods

### CMIP5 and observational data sets

Two sets of core decadal prediction experiments were conducted under CMIP5 (Taylor et al. 2012). The first set was a series of 10-year hindcasts starting approximately in 1960, 1970, 1980, 1990, and 2000. The second was a series of 30-year hindcasts starting in 1960, 1980, and 2005, the last a combined hindcast-forecast. In both sets, AODs (including those due to volcanic eruptions) and solar radiation were prescribed from past observations. Each experiment had a minimum ensemble size of three members. These experiments were somewhat idealistic and exploratory, especially in view of the well-known difficulty of predicting volcanic eruptions.

We used hindcast SST and prescribed AOD data from the HadCM3, CM2.1, CCSM4, and MIROC5 ESMs. Table 1 summarizes major attributes of these models and the CMIP5 decadal hindcast experiments carried out with them. In the CMIP5 hindcast experiments, the CM2.1 used a fully-coupled initialization scheme (Zhang et al. 2007), the MIROC5 used an ocean-only initialization scheme (Tatebe et al. 2012), the CCSM4 used initial ocean and sea ice conditions from a historical forced experiment (Yeager et al. 2012), and the HadCM3 was initialized by relaxation to analyzed ocean and atmosphere observations (Smith et al. 2007). In all CMIP5 experiments, Northern Hemisphere and Southern Hemisphere time series of AOD, based on observations (Ammann et al. (2003) in the NCAR ESM, and Sato et al. (1993) and Hansen et al. (2002) in the other three ESMs), were specified. These data sets provide zonal-average, vertically-resolved AOD for visible wavelengths and column-average effective radii of aerosols (Stenchikov et al. 2006). We also combined hindcast data from the four ESMs in a multi-model ensemble (MME). The MME in this study is the average of the ensemble-average data from each ESM with generally different numbers of ensemble members. In this way, all ESMs are treated equally in forming the MME. We used the Extended Reconstructed SSTs (ERSST; Reynolds et al. 2002) from 1961 to 2010 for evaluating hindcast skills.

**Table 1:** CMIP5 hindcast experiments with Earth System Models used in this study

Model	Institute	Experiment	Ensemble members	SST resolution
CM2.1	NOAA Geophysical Fluid Dynamics Laboratory, USA	Decadal hindcast (1960, 1970, 1980, 1990, 2000)	10	1° (lon.) × 0.34° (lat.) at Eq., and 1° (lat.) at 28° and poleward
HadCM3	Hadley Centre, UK	Decadal hindcast (1060, 1970, 1980, 1990, 2000)	10	1.25° × 1.25°
MIROC5	Atmosphere and Ocean Research Institute (Univ. of Tokyo) National Institute for Environmental Studies, and Japan Agency for Marine-Earth Science and Technology, Japan	Decadal hindcast (1960, 1970, 1980, 1990, 2000)	6	Rotated pole grid ∼ 1.41° (lon.) × 0.79° (lat.)
CCSM4	National Center for Atmospheric Research, USA	Decadal hindcast (1960, 1970, 1980, 1990, 2000)	10	1.25° × 1.25°

### Analysis techniques

We calculated the PDO index from each decadal hindcast experiment by projecting hindcast SSTs from each ESM on the PDO patterns isolated from simulation runs with that ESM (Mehta et al. 2017) to quantify the evolution of the PDO patterns during each 10-year hindcast period. The assumption was that the basic character of the PDO patterns is generally the same in simulation and hindcast experiments conducted with a particular ESM. The TAG and WPWP indices were calculated directly from the hindcast SSTs. These SST indices were calculated by averaging SST anomalies in the WPWP region (20°S to 20°N, 90°E to 180°) for the WPWP index, and in the tropical North (5° to 20°N, 30° to 60°W) and South (0° to 20°S, 30°W to 10°E) Atlantic to calculate the difference between the two to define the TAG index. As mentioned in Mehta et al. (2017), the WPWP SST index has a substantial warming trend. The present analyses were conducted both with and without the warming trend.

For each individual hindcast run of each ESM, a monthly SST anomaly was defined as the departure from its corresponding monthly SST climatology. It is well recognized that ESMs suffer mean biases that are significantly lead-time dependent. To avoid such model drift, the model monthly SST climatology was constructed for each lead time. The hindcast monthly SST anomalies were then derived by subtracting the monthly SST climatology with the same lead time as the hindcast SST.

We estimated deterministic hindcast skill as correlation coefficients and root-mean-square errors (RMSEs) between hindcast and observed DCV indices following Smith et al. (2007), Keenlyside et al. (2008), Pohlmann et al. (2009), Kim et al. (2012), Mehta et al. (2013b), and Gonzalez and Goddard (2016). The skill estimates were calculated with the ensemble-average data as well as data from individual ensemble members from each ESM, and also data from the MME. The individual member skill estimates were used to mark ranges of spread around the skill of ensemble-average hindcasts.

We also estimated the dependence of hindcast skill on the number of ensemble members averaged at various lead times and compared this estimate with an ESM’s ability to predict its own ensemble-average at the same lead times. The former can be called the signal skill and the latter can be called the noise skill. The signal skill’s evolution with respect to the number of ensemble members averaged indicates when (in terms of number of members) the signal skill saturates and a comparison of the two types of skills can be used as an indicator of model performance. In all deterministic skill estimates, following Kim et al. (2012), we used 4-year running windows of observed and hindcast indices to increase the number of samples in each correlation estimates. The t-test (Press et al. 2007) was used to estimate statistical significance of correlation coefficients.

We calculated the Relative Operating Characteristics (ROC) as an indicator of probabilistic hindcast skill. The ROC is commonly used to evaluate the quality of a set of probability forecasts (Swets 1973; Kharin and Zwiers 2003; Landman and Beraki 2012). Gonzalez and Goddard (2016) used the ROC as a probabilistic indicator of skill of CMIP5 models to discriminate between El Niño and La Niña events. The ROC score is relatively independent of forecast initialization; that is, forecast probability and observed relative frequency are independent. Any two forecasts having the same probability ratios, regardless of magnitude, will result in the same ROC score, so the score represents the potential rather than actual predictability. Forecasts having good discriminative ability will show high ROC scores regardless of whether or not probability values are well calibrated. In this paper, the ROC score is shown as the area under the ROC curve (AUC) which is a physical representation of the comparison between the hit rate and false-alarm rate of a set of probabilistic forecasts. The AUC represents the discrimination skill of a set of forecasts with scores above 0.5 indicating potential discrimination skill and a score of 1.0 indicating perfect potential discrimination skill.

In another estimate of probabilistic skill estimate, we calculated probabilities of transition of a DCV index from one phase to another phase (for example, from positive phase PDO^+^ to negative phase PDO^−^) by counting the number of times each phase transition occurred in a given seasonal or annual index time series and then by expressing the number as a percentage of the total number of data points in the index time series. The same approach was followed in calculating transition probabilities of simultaneous phases of more than one DCV phenomena [for example, from the (PDO^+^, TAG^+^) combination to the (PDO^+^, TAG^−^) combination]. For the purpose of assessing hindcast skill of magnitudes of DCV indices, following the definitions of Niño3.4 phases [see, for example, Trenberth (1997)], we defined three states of each index—largest negative value to − ½ times standard deviation (negative), − ½ times standard deviation to + ½ times standard deviation (neutral), and greater than +½ times standard deviation (positive). All index time series were normalized by subtracting the long-term average annual cycle and dividing by standard deviation of the time series.

## Results

### Deterministic skill estimates

Correlation coefficients between hindcast and observed DCV indices, dependent on lead time and number of ensemble members averaged, are described first and they are then compared with correlation coefficients between each ensemble member and the average of the remaining ensemble members. These two groups of coefficients represent signal skill and noise skill, respectively, as mentioned in Sect. 2.2.

**Fig. 1:**
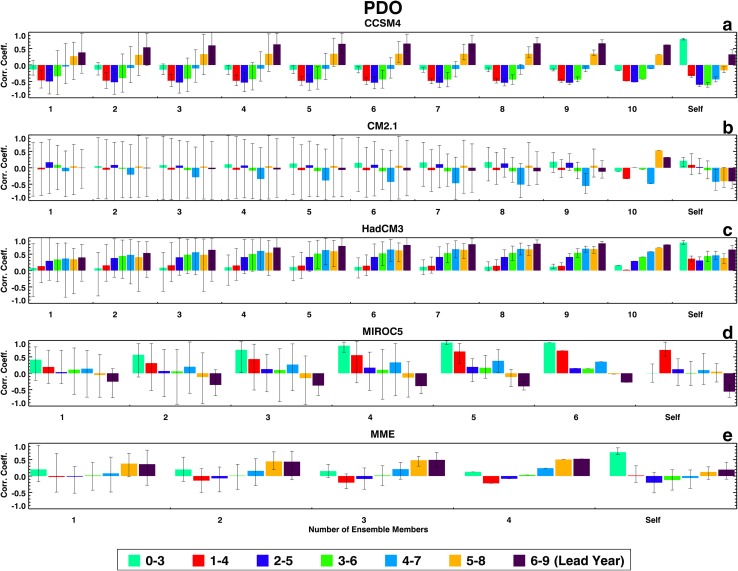
Correlation coefficients between ERSST and hindcast indices of the Pacific Decadal Oscillation (PDO) from 1961 to 2010 in decadal hindcasts made with **a** CCSM4, **b** CM2.1, **c** HadCM3, and **d** MIROC5 ESMs, and **e** MME. Color bars show average correlation coefficients at lead times from 0–3 to 6–9 years, calculated with various combinations of averaged ensemble members. Vertical black lines show the range of coefficients for individual combinations of members. Color bar legend is shown. Self denotes correlation coefficients at lead times from 0–3 to 6–9 years, calculated between each ensemble member and the average of the remaining ensemble members for each model

**Fig. 2:**
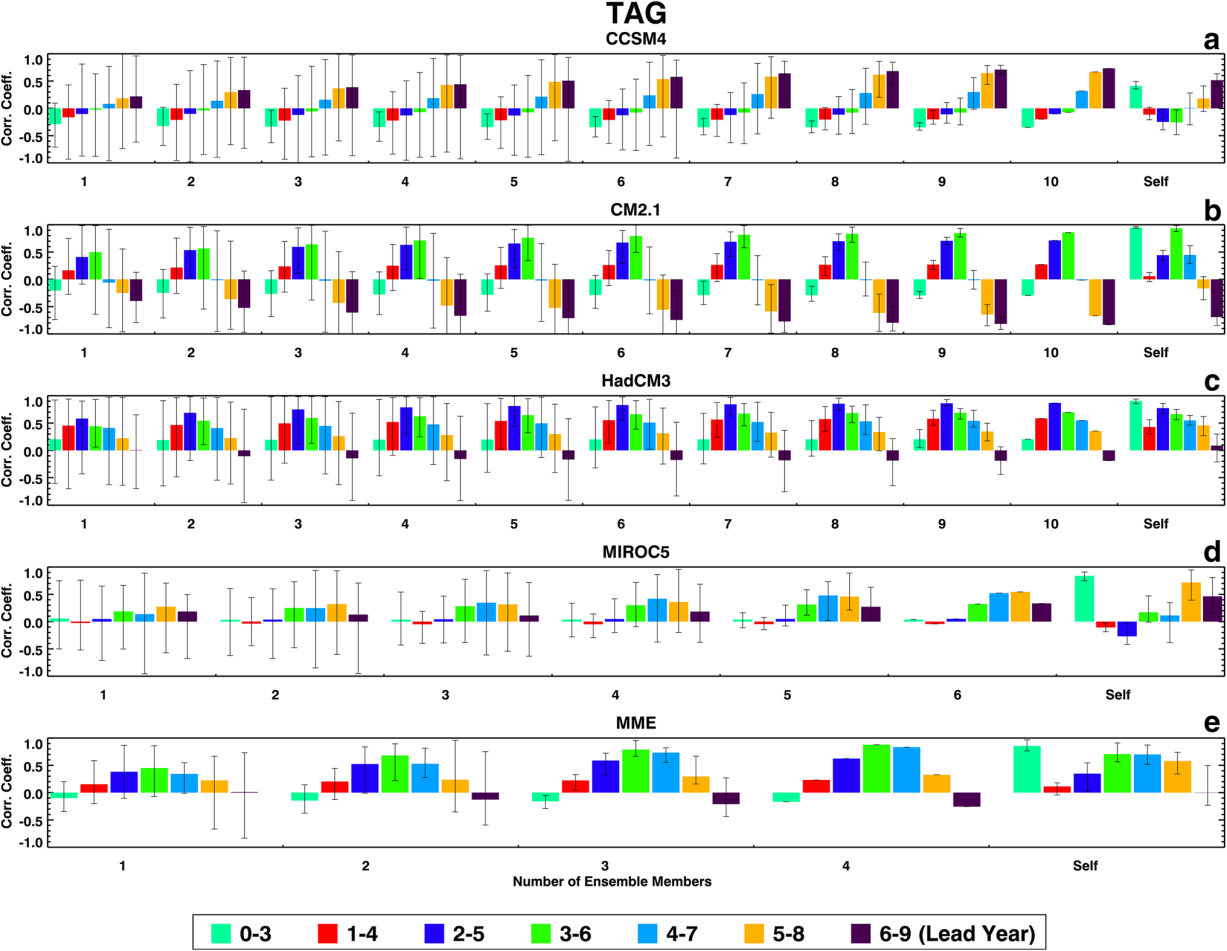
Same as Fig. 1, but for the TAG index

**Fig. 3:**
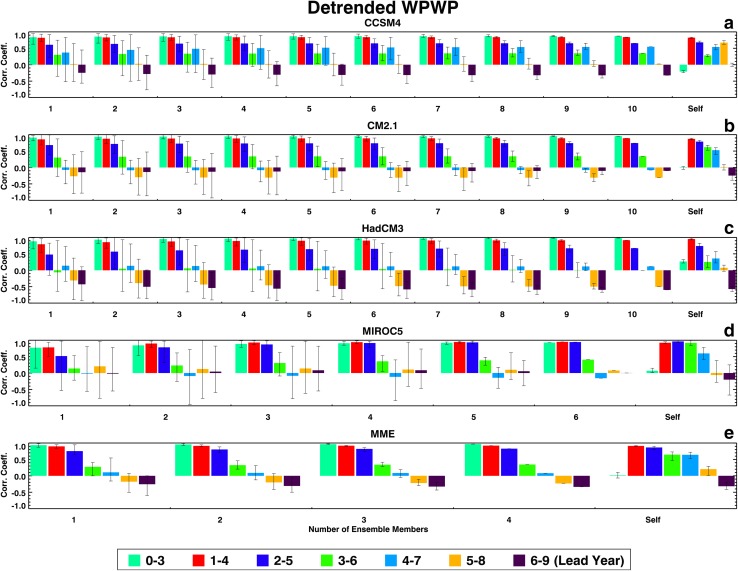
Same as Fig. 1, but for the detrended WPWP index

**Fig. 4:**
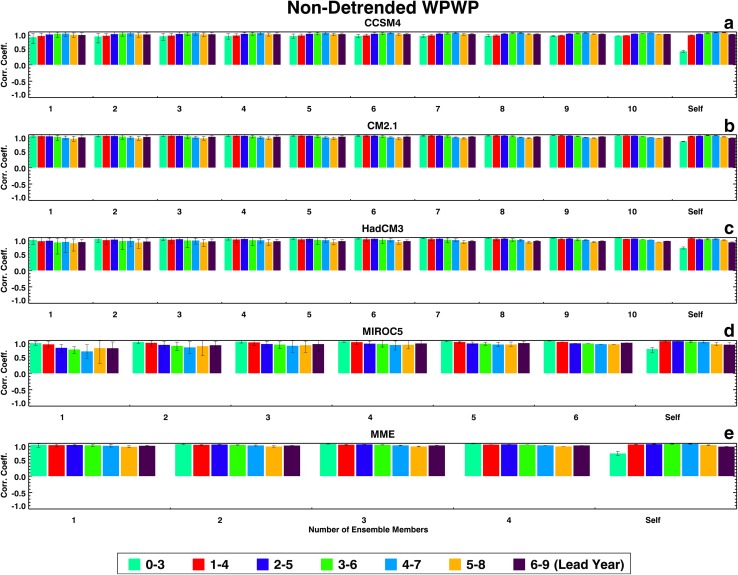
Same as Fig. 1, but for the WPWP index

Figures 1, 2, 3, and 4 show the two groups of correlation coefficients for the PDO, the TAG, detrended WPWP, and non-detrended WPWP indices, respectively. Within each Figure, results for each ESM and the MME are shown in individual panels. Each color bar shows the average of correlation coefficients between an observed index and average index of all unique combinations of ensemble members for one lead time; the black vertical line on each color bar shows the range of minimum and maximum coefficients within an ensemble. Each group of seven bars shows coefficients for each number of ensemble members averaged for seven lead times. The last group, labeled Self, shows correlation coefficients between each ensemble member and the average of the remaining ensemble members. The legend at the bottom of each Figure indicates the lead time in years after initialization of each 10-year hindcast. Figure 1 shows a counter-intuitive progression from no skill to some skill (corr. coeff. 0.5) of PDO prediction in CCSM4 and HadCM3 hindcasts as lead time increases. CM2.1 has no skill at any lead time. MIROC5 shows an increase of skill in the first 4 years after initialization with increasing averaging of ensemble members, with the correlation coefficient reaching almost 1.0 after averaging four ensemble members. The MME correlation coefficients generally shows skill increasing with lead time as in CCSM4 and HadCM3. Figure 1 also shows for all models and the MME decreasing ranges of correlation coefficients between averaged ensemble members and the observed PDO index with increasing numbers of ensemble members. Finally, each model’s ability to predict its own PDO index shows the highest correlation coefficients at the 0–3 years’ lead time for all models except MIROC5 which shows highest correlation coefficient at the 1–4 years’ lead time. These highest self-correlations near the beginning of hindcast runs appear to be consistent with initialization of all ensemble members of a particular model with the same SST data. For each model, the comparison between external index prediction skill and internal index prediction skill is variable. MIROC5 shows a paradoxical behavior as it appears to have a higher skill in predicting observed PDO index compared to the skill in predicting its own ensemble-average PDO index.

The lead time dependence of correlation coefficients for the TAG index (Fig. 2) is model-dependent, with the coefficients increasing with lead time in CCSM4, and peaking in the 2 to 8 years lead time range in the other three models and the MME. They all, however, share the characteristic that the skills increase with increasing numbers of ensemble members averaged with the highest skills close to 0.8 in CM2.1, HadCM3, and the MME. They all also share the characteristic that ranges of coefficients decrease with increasing numbers of ensemble members averaged as the vertical black lines show. Each model’s ability to predict its own TAG index varies with model and lead time, and these self-correlations are generally less than or equal to those for the observed TAG index.

Determinsitic skill dependence of these four ESMs and the MME on lead time for the detrended WPWP index (Fig. 3) is typical of SST index predictability studies [see, for example, Niño3.4 deterministic skill curve in Fig. 6a in Gonzalez and Goddard (2016)]. For all ESMs and the MME in Fig. 3, correlation coefficients between hindcast and observed indices are maximum in the first few years and then decrease as lead time increases. Also, in all ESMs, correlation coefficients increase with increasing numbers of ensemble members averaged, reaching saturation after 4 or 5 members are averaged. The characteristic apparent for the PDO and TAG indices of decreasing ranges of correlations with increasing numbers of ensemble members averaged is also seen in Fig. 3 for the detrended WPWP index. Each model’s ability to predict its own detrended WPWP index, as indicated by the self-correlations, is generally less than or equal to that for the observed detrended WPWP index. In the determinstic skill estimate for the non-detrended WPWP index (Fig. 4), the warming trend overwhelms interannual to decadal variability and the correlation coefficients between observed and hindcast indices are higher than 0.6 to 0.8 in all ESMs and the MME for all lead times. There is a very small decrease in coefficients with increasing lead time in CM2.1, HadCM3, MIROC5, and the MME. Each model’s ability to predict its own WPWP index is also very high as Fig. 4 shows, but it is intriguing to note that these self-correlations are substantially smaller at 0 to 3 years lead times.

Figures 1, 2, 3, and 4 show that ranges of correlation coefficients decrease as larger numbers of ensemble members are averaged to correlate with observed indices and an increasing amount of internal noise is averaged out. It is also evident in these Figures that the skills of ensemble-average hindcasts saturate after 6 to 7 members are averaged. An analysis of the signal skills and noise skills of all ESMs and the MME for all indices shows that if the former skills are larger than the latter skills, the overall hindcast skill is higher. For the PDO index, only the MIROC5 hindcasts show larger signal skill than noise skill in the first few years after initialization. Hindcasts by the other three ESMs and the MME show higher noise skill compared to signal skill in the first few years. For the TAG index, hindcasts by all four ESMs and the MME show higher noise skills than signal skills in the first few years. For both the detrended and non-detrended WPWP indices, hindcasts by all four ESMs and the MME show higher signal skills than noise skills in the first few years, with the result that hindcasts by all ESMs and the MME have high skills for long lead times.

**Fig. 5:**
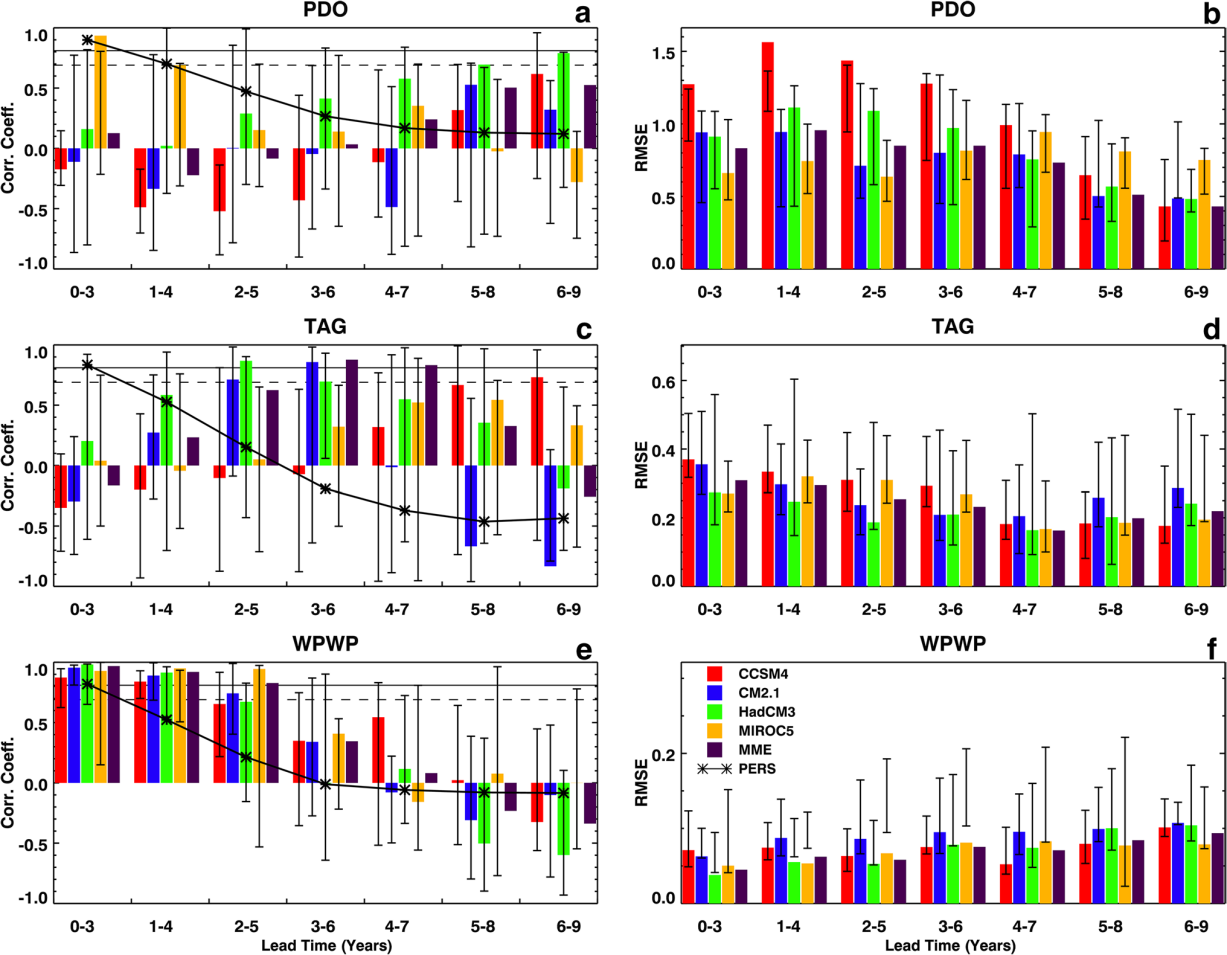
Correlation coefficients and RMSE at various lead times between ERSST and hindcast indices of the PDO, the TAG SST variability, and the detrended WPWP SST variability from 1961 to 2010 in decadal hindcasts made with CCSM4, CM2.1, HadCM3, and MIROC5 ESMs, and the MME. Color bars show correlation coefficients derived from ensemble-average data and vertical black lines show the range of coefficients derived from individual ensemble members. Thick black line connecting stars denotes auto-correlation of each observed index. Dash and solid horizontal lines show 95% and 99% confidence thresholds, respectively. **a** TAG, corr. coeff., **b** TAG, RMSE, **c** WPWP, corr. coeff., **d** WPWP, RMSE, **e** PDO, corr. coeff., and **f** PDO, RMSE

A more conventional (Kim et al. 2012; Gonzalez and Goddard 2016) presentation of deterministic hindcast skills is shown in Fig. 5 in which the ESMs’ skills are also compared with persistence (auto-correlation of observed indices at various lead times). Figure 5 also shows RMSEs of ESMs in hindcasting DCV indices. The color bars in Fig. 5a, c, e show correlation coefficients between the observed DCV index and the ensemble-average hindcast index from each ESM and the MME; ranges of correlation coefficients between the observed index and index from each ensemble member are shown as vertical black lines on each color bar. The ESM associated with each color bar is identified in Fig. 5f. The black line connecting stars in Fig. 5a, c, e is the auto-correlation coefficient of each observed DCV index. Dashed and solid horizontal lines in Fig. 5a, c, e denote 95 and 99% confidence thresholds, respectively. Figure 5b, d, f show RMSEs of hindcasts, where the color bars show the errors in ensemble-average and vertical black lines show ranges of errors in ensemble members.

In Fig. 5, the observed and ensemble-average hindcast PDO (Fig. 5a, b) and TAG (Fig. 5c, d) correlation coefficients are conspicuous by their inclination to peak in the middle or towards the end of the 10 years hindcast runs. This is particularly obvious for the CM2.1, HadCM3, and MME hindcasts of the TAG index at 2 to 7 years lead times, some of which are significant at the 95% or higher level. It is also interesting to note that almost all ESM hindcasts have higher skills than persistence at 2 years and longer lead times even though some of the ESM hindcast skills are not statistically significant. This behavior of the ESMs is seen in the PDO hindcasts (Fig. 5a) at 4 years and longer lead times. The MIROC5 hindcasts of PDO, however, display a more conventional dependence on lead time – largest correlation coefficients at 1 to 4 years lead times and then decreasing with increasing lead time—as Fig. 5a shows. Ranges of correlation coefficients between observed indices and individual ensemble members are very large as Fig. 5a, c show for the PDO and the TAG, respectively. The RMSEs in both PDO (Fig. 5b) and TAG (Fig. 5d) hindcasts are generally smallest at lead times when respective correlation coefficients are largest. As for the correlation coefficients, however, ranges of errors are very large in Fig. 5b, d. Compared to the PDO and TAG hindcast skills, the detrended WPWP index hindcast skill (Fig. 5e) presents a much more conventional dependence on lead times such that the correlation coefficients are largest and larger than persistence skill from initialization to 5 years lead times, and then decrease to zero and below. Corresponding RMSEs of the detrended WPWP index hindcasts (Fig. 5f) are generally smallest when correlations are largest and then increase as the correlations decrease. Ranges of correlation coefficients and RMSEs for the WPWP index are generally smaller when correlation coefficients are largest, especially at lead times closer to initialization. This more conventional presentation of deterministic skill confirms the conclusions drawn from the comparison of signal and noise skills earlier in this Section; that is, MIROC5 has the best hindcast skill among the four ESMs for the PDO index, no one of the ESMs and the MME has TAG hindcast skills, and all four ESMs and the MME have moderate to high hindcast skills for detrended and non-detrended WPWP indices.

### Probabilistic skill estimates

#### Relative operating characteristics

We calculated ROC scores in the form of AUC for hindcasts of each DCV index by each of the four ESMs and the MME as described in Sect. 2.2. The abilities of these hindcasts to discriminate among two phases (positive and negative), and three phases (positive, neutral, and negative) are described here.

**Fig. 6:**
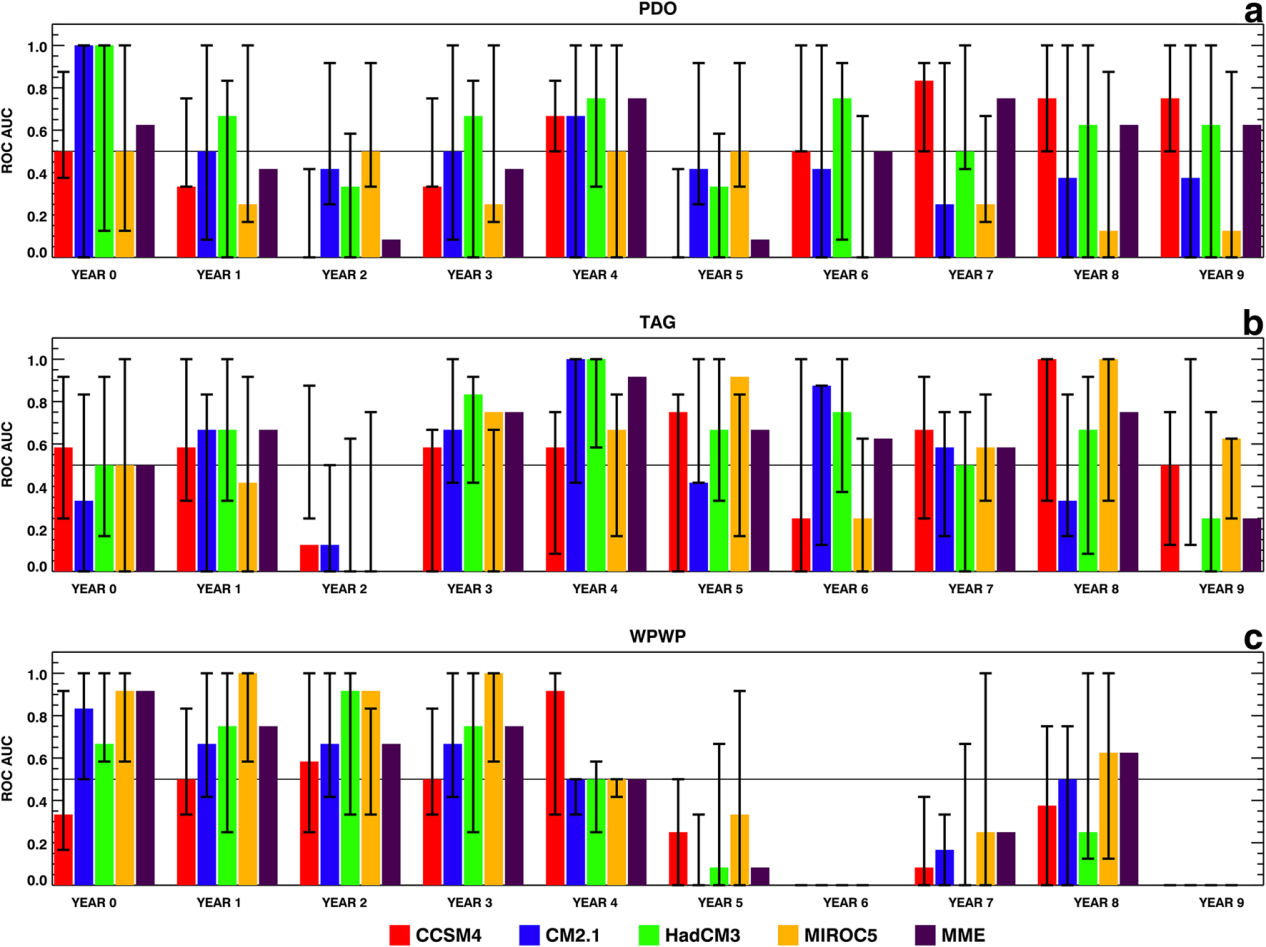
AUC of ROC for probabilistic discrimination between positive and negative phases at various lead times, calculated from ERSST and hindcast indices of the PDO, the TAG SST variability, and the WPWP SST variability from 1961 to 2010 in decadal hindcasts made with CCSM4, CM2.1, HadCM3, and MIROC5 ESMs, and the MME. Color bars show AUC derived from ensemble-average data and vertical black lines show the range of AUCs derived from individual ensemble members. Horizontal black line at 0.5 AUC denotes discrimination threshold. **a** TAG, **b** WPWP, and **c** PDO

At any given lead time, one member within an ESM ensemble may have larger discrimination ability then another member in the same ensemble. Therefore, using each ESM’s ensemble average provides the most reliable hindcast skill estimate for that ESM. Similarly, each ESM’s ensemble average can have higher discrimination skill at any given lead time than another ESM’s ensemble average, therefore, the use of the average of the all ESMs’ ensemble averages, the MME, provides the most reliable indicator of discrimination between or among phases/states. Figure 6 shows the ensemble-average as well as individual ensemble member AUC by lead time in years for positive (above 0) and negative (below 0) phases of each DCV index. For the PDO in Fig. 6a, there is positive discrimination in lead year 0 and, as the lead years increase, there is an increase in potential positive discrimination from year 6 to year 9. Figure 6b shows the AUC for the TAG index for lead times from 0 to 9 years. There is clear discrimination skill for the majority of the lead times except 0 to 2 years. Figure 6c shows the AUC for the detrended WPWP index which shows a more conventional dependence of discrimination skill on lead time, with larger discrimination found in years 0 to 2 and the positive discrimination decreasing with the increase of lead time. Also, positive discrimination skill for WPWP index ends at year 4, but picks up again in year 8 for some of the ESMs and the MME. For all three indices, there are several ensemble members from each ESM which have very high discrimination skill as shown in Fig. 6.

**Fig. 7:**
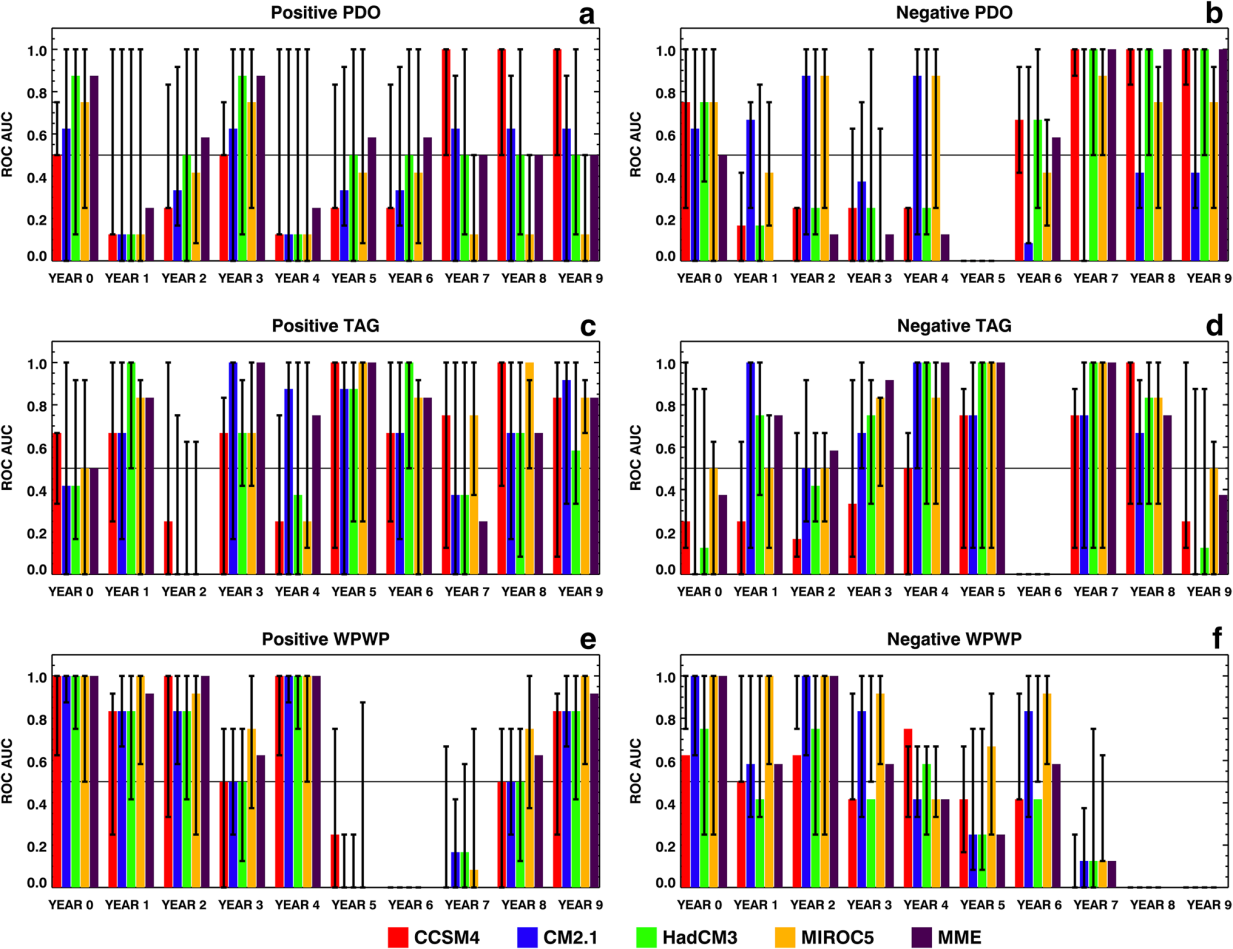
Same as Fig. 6, but for probabilistic discrimination among positive, neutral, and negative phases

We also estimated probabilistic skills to disciminate among three phases of each DCV index. AUC was calculated for positive (greater than or equal to ½ standard deviation above zero), neutral (between − ½ and ½ standard deviations around zero), and negative (less than or equal to − ½ standard deviation below zero) phases for ensemble-average as well as individual member hindcasts. As found by Gonzalez and Goddard (2016) for discrimination skills among three Niño3.4 phases, AUC for the neutral phase of the PDO, TAG, and WPWP indices showed the least amount of potential positive discrimination skill, therefore only the AUCs for discrimination between positive and negative phases are shown here in Fig. 7.

The ensemble-average PDO index AUCs for positive and negative phases (Fig. 7a, b) show no discrimination skill in many of the initial lead years, but show an increase in discrimination skill as the lead years increase for positive (Fig. 7a) and negative (Fig. 7b) phases. After year 5, however, the MME shows positive discrimination skill for positive PDO phase as do CCSM4 and CM2.1 after year 6 (Fig. 7a). The MME and all ESMs except CM2.1 show very high discrimination skill for negative PDO phase after years 6–7 (Fig. 7b). Thus, there are substantial differences in discimination skills for positive and negative PDO phases. For the TAG index (Fig. 7c, d), except in CCSM4 and the MME, there is no discrimination skill for positive phase in the initialization year 0. All four ESMs and the MME show discrimination skill for positive phase in year 1. There is no discrimination in any of the ESMs in year 2, but some or all of the ESMs show moderate to high discrimination skills in the majority of subsequent lead years for positive phase. For negative TAG phase also, there is no discrimination skill in the initialization year in any ESM and the MME, but then the skill increases in subsequent years except in years 6 and 9. Thus, the discrimination skill dependence on lead time is different for positive and negative TAG phases. All four ESMs and the MME show moderate to high discrimination skills for positive phase of the detrended WPWP index (Fig. 7e) from the initialization year to year 4. Then, there is no skill for the next 3 years and the skill increases again in years 8 and 9. For negative phase (Fig. 7f), discrimination skill is high initially, then decreasing in years 3, 4, 5, increasing again in year 6, and then finally declining to no skill. For all three indices, there are several ensemble members from each ESM which have very high discrimination skill for positive and negative phases as Fig. 7 shows.

#### Probabilities of phase transitions

In the second type of estimates of probabilistic hindcast skill, we begin with statistics of occurrence of each DCV phase and of combinations of phases of three DCV phenomena in observed and hindcast DCV indices. Then, observed and hindcast probabilities of transition between positive and negative phases of each DCV phenomenon, and among combinations of phases of three DCV phenomena are described.

The occurrences of each phase, as percent of total number of years, are shown in Table 2 for annual observed DCV indices from 1961 to 2010. Occurrences of individual phases and combinations of phases in ensemble-average indices and the range (minimum to maximum within an ensemble) of occurrences within each ensemble of the four ESMs for the 1961 to 2010 period are also shown in Table 2. If it is assumed that both phases of a DCV index over a multidecadal period have equal probabilities of occurring, then the average occurrence of each phase would be 50% of the period. As Table 2 shows, the occurrence rate is almost 50% for the observed PDO, TAG, and WPWP indices, with small departures from the expected occurrence attributable perhaps to a relatively small sample size. Phase occurrences in three-month average index (December–January–February, DJF; March–April–May, MAM; June–July–August, JJA; September–October–November, SON) data are generally similar (not shown). The corresponding occurrence rates for the ESM hindcast data in Table 2 show that ensemble hindcasts of the three DCV indices have generally comparable occurrence rates with respect to the observed occurrence rates. Ranges of occurrence rates for each ESM’s hindcast ensemble straddle the corresponding ensemble-averages in all except two cases (PDO^+^ and PDO^−^) in MIROC5 hindcasts. Thus, Table 2 shows that all four ESMs hindcast individual DCV phase occurrence rates reasonably accurately.

**Table 2:** Occurrences (% of total number of years) of individual and combination phases of decadal climate variability indices from 1961 to 2010 in observations and hindcasts with individual ESMs and the MME

DCV phases	ERSST	CCSM4		CM2.1		HadCM3		MIROC5		MME	
		Ens.-ave	Member range	Ens.-ave	Member range	Ens.-ave	Member range	Ens.-ave	Member range	Ens.-ave	Member range
PDO^+^	44	44	44–54	42	42–56	48	46–62	40	44–54	44	40–48
PDO^−^	56	56	46–56	58	44–58	52	38–54	60	46–56	56	52–60
TAG^+^	52	50	44–64	54	44–58	48	44–54	52	44–56	56	48–54
TAG^−^	48	50	36–56	46	42–56	52	46–56	48	44–56	44	46–52
WPWP^+^	52	52	42–58	44	44–58	50	40–54	46	46–58	40	44–52
WPWP^−^	48	48	42–58	56	42–56	50	46–60	54	42–54	60	48–56
P^+^T^+^W^+^	8	14	8–24	14	14–26	8	10–26	14	12–24	12	8–14
P^−^T^−^W^−^	12	14	8–22	16	10–24	10	10–26	24	14–22	18	10–24
P^−^T^+^W^+^	20	10	2–16	8	4–14	16	4–16	12	4–8	12	8–16
P^+^T^−^W^−^	12	8	6–14	8	4–16	16	6–22	4	4–12	10	4–16
P^−^T^−^W^+^	18	26	10–24	10	4–18	14	6–18	12	8–20	10	10–26
P^+^T^+^W^−^	18	20	10–18	8	6–16	12	8–14	14	6–10	16	8–20
P^+^T^−^W^+^	6	2	4–16	12	6–22	12	4–20	8	10–20	6	2–12
P^−^T^+^W^−^	6	6	6–16	24	4–18	12	6–20	12	8–22	16	6–24

Some phase combinations of two or all three of the DCV indices are known to be associated with hydro-meteorological (see, for example, Schubert et al. (2004a, b), Mehta et al. (2011b, 2016) and agricultural (Mehta et al. 2012, 2018; Fernandez et al. 2016) impacts in the U.S. Great Plains; impacts on hydro-meteorology, river flows, agriculture, inland water-borne transportation, and hydro-electricity generation in North America; and worldwide impacts on hydro-meteorology, river flows, agriculture, fish captures, and other societal impacts (Mehta 2017). Therefore, it is important to estimate predictability of these phase combinations and their transitions to other combinations. There are eight such combinations (2 phases and 3 DCV indices; 2^3^ = 8) and the theoretical occurrence rate for each phase combination of the three DCV phenomena would be 12.5% if probabilities of all combinations were equal and if the data time series are sufficiently long. These eight combinations are (PDO^+^, TAG^+^, WPWP^+^), (PDO^−^, TAG^−^, WPWP^−^), (PDO^+^, TAG^−^, WPWP^+^), (PDO^+^, TAG^−^, WPWP^−^), (PDO^−^, TAG^+^, WPWP^+^), (PDO^−^, TAG^+^, WPWP^−^), (PDO^+^, TAG^+^, WPWP^−^), and (PDO^−^, TAG^−^, WPWP^+^). In subsequent description of the simultaneous occurrence of two or more DCV phenomena, PDO, TAG, and WPWP are referred to as P, T, and W, respectively, with phases indicated by + or – sign as a superscript. Table 2 shows that the occurrence rates of individual and multiple DCV phases in observed data and ensemble-average ESM hindcasts were generally similar, establishing that the ESM hindcasts represent combinations of DCV phases reasonably well.

**Fig. 8:**
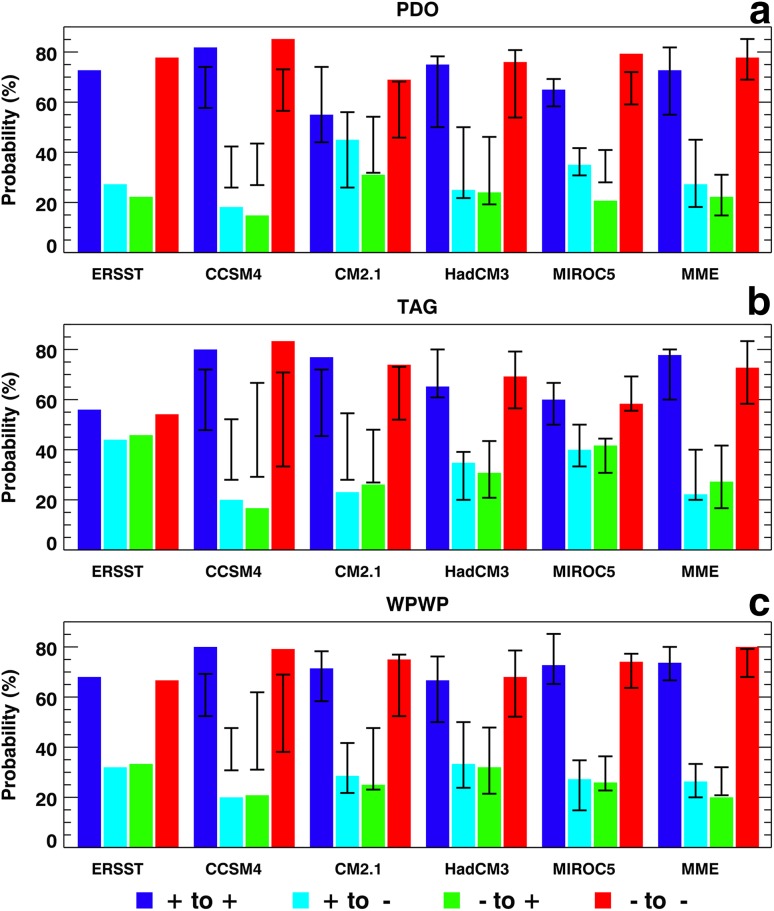
Probabilities of transitions among phases of **a** the PDO, **b** the TAG SST variability, and **c** the WPWP SST variability from 1961 to 2010 in ERSST data, and in decadal hindcasts made with CCSM4, CM2.1, HadCM3, and MIROC5 ESMs, and the MME. For the model data, color bars show probabilities derived from ensemble-average data and vertical black lines show the range of probability derived from ensemble members

Next, the probabilities of transition from the phase in one year to either of the two possible phases of individual DCV indices in the next year in the observed and hindcast annual data were estimated and are shown in Fig. 8. Ranges of within-ensemble transition probabilities in the ESM hindcasts are also shown in Fig. 8 as vertical black line on each color bar. These ranges were calculated from individual ensemble members for each ESM and the MME. For the PDO phases (Fig. 8a), there is an overwhelming tendency for same-phase transitions, or persistance, of PDO from one year to the next. Ensemble-average hindcasts by all ESMs and the MME generally show this tendency in Fig. 8a. Even including the ranges of probabilities for each ESM in the comparison, the higher probabilities of same-phase transitions are clearly evident. There are some seasonal variations in probabilities in the observed, ESM, and MME data, with the same-phase PDO transitions most probable in June–July–August.

Figure 8b shows that TAG phases are less persistent than PDO phases in observed data and their transition probabilities are approximately equal, although same-phase transitions have higher probabilities. TAG phases in the four ESMs and the MME are more persistent as indicated by considerably larger same-phase transition probabilities for annual data in Fig. 8b. Consequently, opposite-phase transition probabilities are much lower in the individual ESM and MME hindcasts. As for the PDO and TAG phases, same-phase transition probabilities of WPWP phases in the observed annual data (Fig. 8c) are much higher compared to the opposite phase transition probabilities. The same-phase transition probabilities in ensemble-average annual data from the four ESMs and the MME (Fig. 8c) are at least as high as the probabilities in the observed data even when the within-ensemble ranges are included in the comparison. Consequently, opposite phase transition probabilities in the four ESMs and the MME are equal to or lower than those in the observed data. Thus, Fig. 8 shows that probabilities of same-phase transitions from one year to the next are considerably larger than opposite-phase transitions for PDO and WPWP phases in observed data and ensemble-average ESM and the MME hindcasts, except in the CM2.1 hindcasts where the differences among probabilities of PDO phase transitions are much smaller. Probabilities for TAG phases are almost the same in the observed data, but in the ensemble-average ESM and MME hindcasts the same-phase transition probabilities are much larger than the opposite-phase probabilities.

**Fig. 9:**
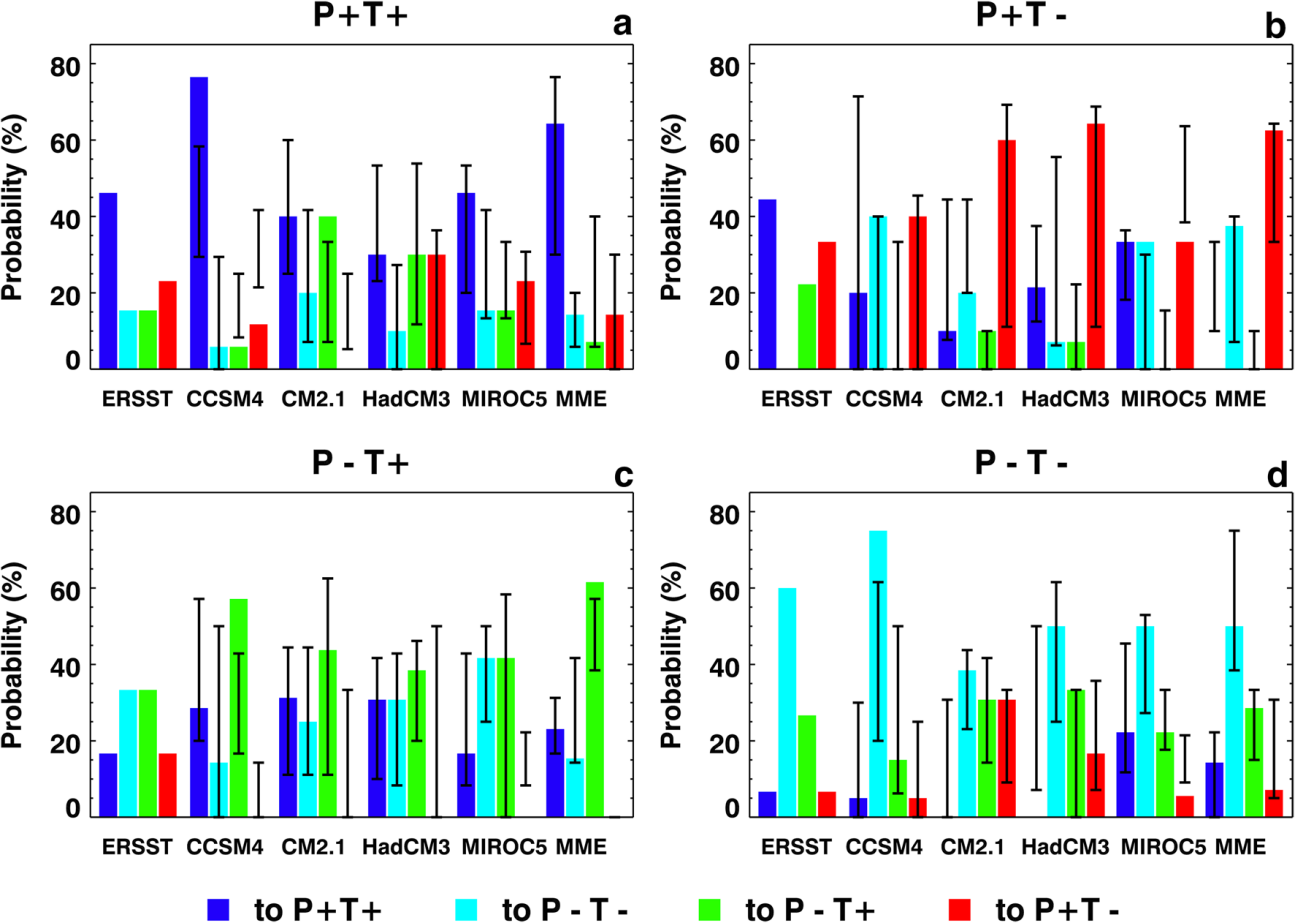
Probabilities of transitions among combined phases of the PDO and the TAG SST variability from 1961 to 2010 in ERSST data, and in decadal hindcasts made with CCSM4, CM2.1, HadCM3, and MIROC5 ESMs, and the MME. **a** PDO^+^, TAG^+^; **b** PDO^+^, TAG^−^; **c** PDO^−^, TAG^+^; and **d** PDO^−^, TAG^−^. For the model data, color bars show probabilities derived from ensemble-average data and vertical black lines show the range of probability derived from ensemble members

Next, we consider transition probabilities among combinations of phase of two DCV phenomena, the PDO and TAG variability. There are four possible combinations of phenomena and phases—(P^+^, T^+^), (P^−^, T^−^), (P^+^, T^−^), and (P^−^, T^+^)—and the theoretical transition probability for each transition would be 25% if the transitions occur randomly; that is, there would be equal probabilities of a transition to any of the four combinations. The actual transition probabilities of combined PDO and TAG phases are shown in Fig. 9 as four color bars, one for each phase combination, for observed and ESM—including MME—data sets. Ranges of within-ensemble transition probabilities in the ESM hindcasts are also shown in Fig. 9 as vertical black lines superimposed on each color bar. A general tendency of all four combinations in the observed and ensemble-average ESM and MME indices to remain in the same combination is obvious in Fig. 9, including when the ranges of ensemble member results are included, although there are cases in which probabilities are higher for transitions to other combinations (for example, (P^+^, T^+^) in CM2.1 and HadCM3). This general observation implies that ensemble-average results may be reliable enough for actual prediction of phase combinations at one to two years lead times. Details show, however, that there are very large ranges of transition probabilities for some combinations, pointing to the need for ensembles and ensemble averaging, including MME averaging.

#### Phase hindcast skills

As mentioned in Sect. 2.1, the decadal hindcast experiments were initialized once (in the 0th year—1960, 1970, etc.) every 10 years. The phase hindcast skills for the PDO, TAG, and the WPWP indices in the first and second year after initialization are described here. For both the first and second years, we analyzed the accuracy of phase hindcast using data from annual-average and ensemble-average hindcasts as well as from all individual members of each ensemble. The results for both first and second years are shown in Table 3 for the PDO. The ensemble averages from the ESMs and the MME hindcast the PDO phase in the first year after initialization correctly in all five decades, except for CM2.1 in 1961. The second year phase hindcast by ensemble averages was correct for three ESMs (CCSM4, CM2.1, and MIROC5) and the MME in 1982. In other decades, however, fewer ensemble averages from individual ESMs hindcast the PDO phase correctly. The ensemble-average MME hindcast of the PDO phase in the second year was correct in 1982, 1992, and 2002. Table 3 also shows that first year phase hindcasts of the PDO index by individual members of each ensemble were correct for the largest number of members of CCSM4 ensembles in all five decades, followed by MIROC5 and the MME. It is obvious that the success rate or skill of phase prediction decreases from first year to second year for CCSM4, CM2.1, and HadCM3, but the second-year phase prediction skill of MIROC5 hindcasts is 100% in four of the five decades. It is also interesting to note that a correct hindcast of first-year PDO phase appears to be a necessary but not a sufficient condition for a correct hindcast of second-year phase.

**Table 3:** One- and two-year phase prediction skill in decadal hindcasts of the PDO in each decade from 1961 to 2010

Earth system model (ensemble members)	1961–1970		1971–1980		1981–1990		1991–2000		2001–2010	
	1961 (−)	1962 (−)	1971 (−)	1972 (−)	1981 (+)	1982 (+)	1991 (−)	1992 (+)	2001 (−)	2002 (-)
CCSM4 (10)	**10**	0	**9**	0	**9**	**10**	**10**	**7**	**9**	**8**
CM2.1 (10)	3	0	**5**	**6**	**8**	**8**	**5**	7	**4**	3
HadCM3 (10)	**3**	2	**1**	5	**5**	6	**6**	3	**10**	**7**
MIROC5 (6)	**4**	**5**	**6**	**3**	**4**	**5**	**3**	**6**	**6**	2
MME (36)	**20**	7	**21**	14	**26**	**29**	**24**	**23**	**29**	**20**

In parentheses after the ESM name are shown the number of ensemble members for each ESM. The phase of the observed PDO index (−/+) in first and second year of each decade is shown in parentheses after each year. Bold numbers denote correct phase prediction by the annual-average, ensemble-average hindcast by each ESM and the MME, and the numbers denote how many members of each ensemble also hindcast the phase correctly

As for the PDO index, MIROC5 performs better than the other three ESMs and the MME for the second year prediction of the TAG index also (Table 4) with correct phase prediction in four out of five decades. CCSM4, CM2.1, and the MME are next with three correct predictions of second-year TAG phase out of five decades, and HadCM3 has correct prediction of second-year phase in two out of five decades. Unlike for PDO predictions, however, a correct first-year prediction of the TAG phase does not appear to be a necessary condition for a correct second-year phase prediction. Of the three DCV indices, first- and second-year hindcasts of the detrended WPWP index are correct in the majority of the ESM-decade combinations (Table 5). In 1961, 1981, and 2001, ensemble-average WPWP index hindcasts by all four ESMs and the MME are correct for the first year after initialization. In 1962, 1992, and 2002, second-year phase hindcasts are also correctly made by ensemble-average WPWP indices by all four ESMs and the MME. It is also remarkable that when the first/second year phase of the WPWP index is correctly hindcast by the ESMs and the MME, almost all members of the corresponding ensembles also hindcast the phase correctly.

**Table 4:** Same as Table 3, but for the TAG index

ESM (ensemble members)	1961–1970		1971–1980		1981–1990		1991–2000		2001–2010	
	1961 (+)	1962 (+)	1971 (−)	1972 (−)	1981 (+)	1982 (+)	1991 (−)	1992 (+)	2001 (−)	2002 (−)
CCSM4 (10)	4	**10**	0	5	**10**	4	0	**6**	**10**	**9**
CM2.1 (10)	**7**	**10**	1	4	2	2	**6**	**7**	**7**	**6**
HadCM3 (10)	**8**	**7**	1	4	**6**	2	1	5	**10**	**5**
MIROC5 (6)	**4**	**6**	**4**	**3**	**5**	0	2	**4**	**6**	**4**
MME (36)	**23**	**33**	6	16	**23**	8	9	**22**	**33**	**24**

**Table 5:** Same as Table 3, but for the detrended WPWP index

ESM (ensemble members)	1961–1970		1971–1980		1981–1990		1991–2000		2001–2010	
	1961 (+)	1962 (+)	1971 (−)	1972 (−)	1981 (+)	1982 (−)	1991 (−)	1992 (−)	2001 (+)	2002 (+)
CCSM4 (10)	**10**	**10**	2	0	**9**	2	**10**	**10**	**7**	**10**
CM2.1 (10)	**10**	**10**	**8**	0	**10**	1	6	**10**	**8**	**10**
HadCM3 (10)	**10**	**10**	**9**	3	**9**	**5**	7	**10**	**7**	**8**
MIROC5 (6)	**5**	**5**	**6**	**3**	**6**	1	2	**6**	**5**	**6**
MME (36)	**35**	**35**	**25**	6	**34**	9	**25**	**36**	**27**	**34**

Observed and hindcast WPWP indices were detrended before calculation of prediction skill

### Roles of volcanic eruptions and internal variability in phase transitions

Sustained transitions in phases of the PDO, and the TAG and WPWP SST variabilities in observed and ensemble-average hindcast indices were visually identified. The phase transitions occurred over many months to 1 to 3 years and there is some subjectivity in the choice of selected transitions. The observed and hindcast phase transitions were also compared with major volcanic eruptions at low latitudes as represented in AOD time series and other publicly available information. Major eruptions are defined here as Volcanic Explosivity Index (VEI; Newhall and Self 1982) 4 or greater. There were four such low-latitude eruptions in the 1961 to 2010 period: (1) February to May 1963, Mount Agung, Bali, Indonesia, VEI 5; (2) October 1974 to early 1975, Volcan de Fuego, Guatemala, VEI 4; (3) March to April 1982, El Chichón, Mexico, VEI 5; and (4) June 1991, Mount Pinatubo, Philippines, VEI 6. The following questions were addressed to visually identify roles of volcanic eruptions and internal variability in DCV phase transitions.

1. Are there phase transitions in observed and hindcast DCV indices which are physically consistent with volcanic eruptions as represented by AOD changes?
2. Are there phase transitions in observed DCV indices which are also hindcast by the ESMs, but are not associated with AOD changes?
3. Are there phase transitions in observed DCV indices which are in simulations and initialized hindcasts? Are they associated with AOD changes?
4. What is the impact, if any, of initialization on phase transition events and on overall hindcasts?
5. Is a correctly simulated response of a DCV index to a major volcanic eruption a pre-requisite for a correct hindcast in response to the same event?

In the following description of results, positive to negative phase transitions are referred to as PTN and negative to positive phase transitions are referred to as NTP.

#### Pacific decadal oscillation phase transitions

There were 14 PDO phase transitions between 1961 and 2010 in the observed data, with each phase persisting for many months to many years. There were two types of phase transitions in the observed PDO index—transitions associated with internal ocean–atmosphere dynamics and those associated with AOD changes associated with volcanic eruptions. From these visual inspections, summary answers to the questions posed are: (1) Three PDO phase transitions are associated with volcanic eruptions in both observed and hindcast indices in all ESMs and the MME (Mount Agung, Volcan de Fuego, Mount Pinatubo), except for the absence of the Volcan de Fuego transition in CCSM4; (2) All ESMs’ hindcasts capture phase transitions not associated with AOD changes in varying numbers, such correct transitions in an ESM’s hindcast vary from two to six; (3) The Mount Agung and Mount Pinatubo transitions are in simulations with all four ESMs and the MME also, but the sizes of the simulated changes vary among the ESMs and the MME (Mehta et al. 2017); (4) The 1976–1977 NTP transition is simulated by CM2.1, HadCM3, and CCSM4 to some extent, which suggests the intriguing possibility that perhaps coupled ocean–atmosphere response to the 1974–1975 Volcan de Fuego volcanic eruption resulted in the 1976–1977 NTP transition; this transition is present, but without the full range of PDO index change, only in ensemble-average hindcasts by CM2.1 and the MME initialized in 1970. Thus, initialization appears to have interfered with this NTP transition in HadCM3 and CCSM4 ESMs if indeed it was caused as a response to the Volcan de Fuego eruption; and (5) a correctly simulated response to AOD changes associated with volcanic eruptions does not appear to be a pre-requisite for an ESM to successfully hindcast the PDO response to the same forcing change.

#### Tropical Atlantic SST gradient phase transitions

There were 9 TAG phase transitions between 1961 and 2010 in the observed data, each of which persisted in positive or negative phase for many months to many years. From visual inspections, summary answers to the questions posed are: (1) There are no TAG phase transitions in hindcast data which are also in observed data and are associated with AOD changes; (2) There are no TAG phase transitions in hindcast data which are also in observed data, but are not associated with AOD changes; (3) some of the TAG phase transitions which are in observed data are simulated by some of the ESMs and the MME, but they are not hindcast by any ESM; (4) initialization appears to have interfered with the ESMs’ hindcasting the correct response to major volcanic eruptions; and (5) since no TAG phase transition is hindcast by any of the ESMs, its correct simulation by the same ESM does not appear to be important. With respect to a lack of the TAG index’s response to volcanic eruptions in these ESMs, it is possible, as Swingedouw et al. (2015) found, that the response of some ESMs to Mount Agung-like, low-latitude volcanic eruptions on North Atlantic Ocean circulation and temperature is delayed by several years to a decade. Therefore, the response of the Atlantic climate in general and the TAG phenomenon in particular in these ESMs to low-latitude volcanic eruptions in the simulation and hindcast modes needs to be studied further via controlled experimentation with these ESMs.

#### West Pacific warm pool variability phase transitions

There were 9 phase transitions in the WPWP SST index from 1961 to 2010 in the observed data, with each phase persisting for many months to many years. As in the cases of PDO and TAG phase transitions, there are two types of transitions in WPWP index; one group associated with internal ocean–atmosphere dynamics and the other associated with AOD changes associated with volcanic eruptions. From these visual inspections and a comparison with simulations by these four ESMs (Mehta et al. 2017), summary answers to the questions posed are: (1) There are four phase transitions associated with the four major volcanic eruptions in the observed WPWP index which were generally correctly hindcast by the four ESMs and the MME; (2) all ESMs’ ensemble-average hindcasts capture transitions not associated with volcanic eruptions in 1993–1994 and 1994–1996 to varying degrees; (3) simulations with all four ESMs and the MME capture the 1963–1964, 1981–1982, and 1991–1993 phase transitions associated with volcanic eruptions in the WPWP SST index. Simulations with MIROC5 and HadCM3 also capture the 1973–1976 phase transition; ranges of simulated transitions, however, vary among the ESMs and the MME; (4) The impact of initialization appears to reinforce the four transitions associated with volcanic eruptions and correct hindcasts of two additional transitions not associated with volcanic eruptions. The latter two, however, are also present in simulations with all four ESMs and the MME, so perhaps there is another radiative forcing (not AOD changes) driving these two transitions. It is also interesting to note that simulations show warming trend in the WPWP SST index continuing after 1996 which is not captured by any of the ESMs’ hindcasts; and (5) a correct simulation of the WPWP index’s response to volcanic eruptions appears to be a pre-requisite to correct hindcasts in response to the same events.

## Summary and discussion

We estimated deterministic and probabilistic hindcast skills of the PDO, the TAG SST variability, and the WPWP SST variability in ensembles of decadal hindcasts made with the CCSM4, CM2.1, HadCM3, and MIROC5 ESMs—and the MME formed from these ESM hindcasts—from 1961 to 2010. We also estimated positive and negative phase occurrence rates, phase transition probabilities, and one-year and two-year phase predictability. We then conducted case studies of individual, sustained phase transitions in the ensembles of decadal hindcasts in order to attribute the transitions to volcanic eruptions or internal ocean–atmosphere variability. We found that:

- Deterministic skill depends on the number of ensemble members averaged up to 6–7 members, then the skill saturates. Deterministic skills to hindcast an observed DCV index and to hindcast an ESM’s internally-generated DCV index - which can be called signal skill and noise skill, respectively – vary among the ESMs and also vary among lead times after initialization. In all ESMs and the MME for all DCV indices, higher deterministic skill hindcasts have larger signal skills than noise skills in the first 4 years. Only MIROC5 hindcasts of the PDO index have higher signal skill than noise skill in the first 4 years, resulting in a higher overall determinstic skill. TAG hindcasts by all ESMs and the MME have higher noise skills than signal skills in the first 4 years. Hindcasts of detrended and non-detrended WPWP indices by all ESMs have higher signal skill than noise skill in the first 4 years; both indices have high overall skills. Deterministic skills of the PDO and TAG improve in the CCSM4, HadCM3, and CM2.1 at lead times longer than 4 years when signal skills become at least equal to noise skills. Ranges of individual member correlation coefficients and RMSEs for the all three DCV indices are generally smaller when correlation coefficients are largest, especially at lead times closer to initialization.
- Probabilistic skill, as represented by the ROC AUC, was estimated for the ability of the decadal hindcasts to discriminate among positive, neutral, and negative phases of the three DCV indices. There is no discrimination skill in hindcasts of the PDO index in some of the initial years, but show an increase in discrimination skill as the lead years increase for both positive and negative phases. For the TAG index, there is no discrimination skill in the first year after initialization, but the majority of subsequent lead years show high discrimination skills. For the WPWP index, there is high discrimination skill in the initial years, with decreasing discrimination skill at longer lead years. There is no discrimination skill for the neutral phase of all three indices in any of the ESM’s hindcasts. The general consistency of the dependence of deterministic and probabilistic skills on lead time increases confidence in both types of skill estimates.
- Ensemble-average hindcasts of the three DCV indices made with the four ESMs and the MME have generally comparable phase occurrence rates with respect to observed rates. There is a moderate to high probability of phase persistence or same-phase transitions of PDO and WPWP phases from one year to the next in observed data and also generally in the ensemble-average ESM hindcasts, whereas the same-phase transition probability of TAG phases is moderate. There is a general tendency of all four combinations of PDO and TAG phases in the observed and ensemble-average ESM indices to remain in the same combination for at least two years, although there are cases in which probabilities are higher for transitions to other combinations.
- Ensemble-average and most of ensemble members of MIROC5 hindcasts correctly predict PDO phases one and two years after initialization in all five decades from 1961 to 2010. Prediction success rate decreases from the first year to the second in CCSM4, CM2.1, and HadCM3 hindcasts. Ensemble-average and most of ensemble members of the MME hindcasts correctly predict PDO phases one and two years after initialization after 1980; they correctly predict only the first-year PDO phase in 1960s and 1970s.
- Major, low-latitude volcanic eruptions in 1963 (Mount Agung), 1974–1975 (Volcan de Fuego), 1981–1982 (El Chichón), and 1991–1992 (Mount Pinatubo) are associated with sustained phase transitions of DCV indices in observed data and in some of the ensemble-average decadal hindcasts. Three of the four major volcanic eruptions were associated with PDO phase changes in observed data and almost all hindcasts. The WPWP index phase changes associated with all four eruptions were hindcast by all ESMs and the MME. In contrast, no one of the nine TAG phase transitions in observed data was present in any hindcast. Hindcasts from some of the ESMs and the MME show approximately correct phase transitions in the absence of AOD changes also, implying that the initialization of the ESM hindcasts with observed data is beneficial in predicting phase transitions of DCV indices.

Before these results are discussed further, it must be mentioned that there are several shortcomings of these ESMs and decadal hindcast/forecast experiments conducted with them as mentioned in Sect. 1.2. Additionally, the four ESMs selected for the present study were initialized with different techniques. In spite of these and other shortcomings such as the inclusion of future volcanic eruptions in decadal hindcasts, the results of the analyses presented in this paper shed considerable light on prospects for future predictions of DCV indices and their usability for impacts prediction.

The results presented in this paper indicate that the persistance and phase transition probability statistics of DCV indices and their predictability by the ESMs, and also perhaps long-term evolutions, can be exploited for prediction of these indices’ possible impacts on hydro-meteorology, streamflows, agriculture, and other societal sectors. For example, the PDO, the TAG, and the WPWP SST indices are associated with precipitation, temperature, river flows, and crop yields in the Missouri River Basin (MRB)—the largest river basin in the U.S. and a major “bread basket” of not only the U.S. but also of the world. Positive phase of the PDO and negative phase of the TAG increase wetness and river flows, and negative phase of the PDO and positive phase of the TAG decrease wetness and river flows in the MRB. As a result, as described in the Introduction, a successful prediction of phases of these phoenomena one year in advance can be very useful to the agricultural economy of the MRB. Another example of usefulness of successful phase prediction is in predicting Mississippi River flow variations associated with the PDO and the TAG a year or longer in advance. Over 500 million tons of grain and other cargo are carried by barges on the Mississippi River annually, and both low-flow and high-flow conditions associated with the PDO and the TAG make substantial impacts on the water-borne commerce economy of the Mississippi River. Both these applications of DCV phase prediction are under way in collaboration with stakeholders in these two river basins. At longer than one or two years’ lead times, MIROC5 ESM appears to have the best overall performance from this group of ESMs. This reasonably encouraging performance of MIROC5 in hindcasting the PDO and WPWP indices over the 1961 to 2010 period was the reason for using the MIROC5 data to hindcast decadal hydrologic cycles in seven countries of southern Africa by Mehta et al. (2014) and can be used for other applications also.

Although it is (almost) impossible to predict volcanic eruptions of any explosivity, it is instructive that AOD changes associated with major volcanic eruptions were included in the CMIP5 hindcast experiments. As the results show, the four ESMs and the MME appear to respond accurately to varying degrees to the eruption-associated AOD changes, and the hindcasts of the PDO and WPWP indices show phase transitions and subsequent evolutions of the DCV indices comparable to those in observed indices for several months to several years in some cases. Therefore, these hindcast results give encouragement for the use of these and other ESMs for multi-year prediction initialized soon after a major volcanic eruption occurs. Volcanic AOD changes appear to cause damped oscillations in the DCV indices in some cases over several years, which might extend predictability of these indices beyond the immediate effects of AOD changes. These impacts of eruption-associated AOD changes on DCV indices imply that volcanic eruptions can influence global atmospheric dynamics and climate not only directly via interactions between ejected material in the atmosphere and short- and long-wave radiations, but also via influencing DCV phenomena’s impacts on global climate.

This surprising ability of this group of ESMs to hindcast an observed DCV index better than their ability to hindcast their own index at various lead times is truly enigmatic and further research is necessary to understand causes and implications of such model behavior. Such enigmatic behavior of signal and noise skills for the TAG index, and non-response of TAG hindcasts to major volcanic eruptions are consistent with a similar behavior of a global atmospheric model to predict the North Atlantic Oscillation index in response to observed SSTs (Mehta et al. 2000), indicating that there is something fundamentally incorrect about the Atlantic region’s responses to internal and external forcings in atmosphere-alone and coupled Earth System models.

Also enigmatic are the differences in behaviors of this group of ESMs in simulation and prediction modes. Simulations of the three DCV phenomena with the same ESMs and the MME (Mehta et al. 2017) show that while these ESMs simulate the PDO’s attributes (spatial pattern, annual cycle, and variability timescales) reasonably well, the ESMs only simulate the annual cycle and variability timescales of the WPWP SST variability reasonably well and the WPWP’s spatial pattern is very poorly simulated by the ESMs and the MME. In the case of the TAG SST variability, simulation results show that while the spatial pattern simulation by the ESMs and the MME is approximately correct, the annual cycle and variability timescales are simulated very poorly. As results presented in this paper show, however, the WPWP index has the best deterministic and probabilistic hindcast skills, only MIROC5 shows reasonably high hindcast skills of the PDO index, and all of the ESMs show poor TAG hindcast skills. These differences between simulations and predictions of the same phenomenon by the same ESMs have serious implications not only for the prediction of impacts of these phenomena on global climate and society, but also about the simulation and prediction/projection of future climate change and its impacts. This is especially true about the WPWP since it is the largest heat source for driving global atmospheric circulations. Despite these problems, as the encouraging results in this and other cited papers indicate, the day may not be very far in the future when some aspects of DCV information are skillfully predicted and routinely used in agriculture and water resource managements, and other societal sectors.
